# Spatially Distributed Effects of Mental Exhaustion on Resting-State FMRI Networks

**DOI:** 10.1371/journal.pone.0094222

**Published:** 2014-04-04

**Authors:** Fabrizio Esposito, Tobias Otto, Fred R. H. Zijlstra, Rainer Goebel

**Affiliations:** 1 Department of Medicine and Surgery, University of Salerno, Baronissi (Salerno), Italy; 2 Department of Cognitive Neuroscience, Maastricht University, Maastricht, The Netherlands; 3 Department of Work and Social Psychology, Maastricht University, Maastricht, The Netherlands; 4 Department of Vision and Cognition, Netherlands Institute for Neuroscience, Amsterdam, The Netherlands; University of Maryland, College Park, United States of America

## Abstract

Brain activity during rest is spatially coherent over functional connectivity networks called resting-state networks. In resting-state functional magnetic resonance imaging, independent component analysis yields spatially distributed network representations reflecting distinct mental processes, such as intrinsic (default) or extrinsic (executive) attention, and sensory inhibition or excitation. These aspects can be related to different treatments or subjective experiences. Among these, exhaustion is a common psychological state induced by prolonged mental performance. Using repeated functional magnetic resonance imaging sessions and spatial independent component analysis, we explored the effect of several hours of sustained cognitive performances on the resting human brain. Resting-state functional magnetic resonance imaging was performed on the same healthy volunteers in two days, with and without, and before, during and after, an intensive psychological treatment (skill training and sustained practice with a flight simulator). After each scan, subjects rated their level of exhaustion and performed an N-back task to evaluate eventual decrease in cognitive performance. Spatial maps of selected resting-state network components were statistically evaluated across time points to detect possible changes induced by the sustained mental performance. The intensive treatment had a significant effect on exhaustion and effort ratings, but no effects on N-back performances. Significant changes in the most exhausted state were observed in the early visual processing and the anterior default mode networks (enhancement) and in the fronto-parietal executive networks (suppression), suggesting that mental exhaustion is associated with a more idling brain state and that internal attention processes are facilitated to the detriment of more extrinsic processes. The described application may inspire future indicators of the level of fatigue in the neural attention system.

## Introduction

Functional magnetic resonance imaging (fMRI, [1]) in the absence of experimental tasks and behavioral responses, performed with the subject in a relaxed resting state (RS-fMRI) allows measuring the amount of spontaneous blood-oxygen-level-dependent (BOLD) signal synchronization within and between multiple regions across the entire brain [2]. In fact, RS-fMRI activity is characterized by low frequency (0.01–0.1 Hz) BOLD signal fluctuations, which are topologically organized as multiple spatially distributed functional connectivity networks called resting-state networks (RSNs) [3], [4], [5], [6], [7] and recent high temporal resolution RS-fMRI studies have found a similar organization even at frequencies above 0.1 Hz (see, e.g., [8]). As the RSN constituent regions partially or totally overlap with typical brain activations induced by perceptual and cognitive tasks [9], [10], the study of one or more RSNs has allowed a system-level functional description of several mental processes and the characterization of the associated brain status. More recently, by repeating RS-fMRI scans under different experimental conditions, it has been possible to relate these processes to (and manipulate by) externally modifiable factors, such as different pharmacological treatments or psychological experiences [11], [12], [13], [14], [15].

Independent component analysis (ICA) [16], if applied to whole-brain fMRI time-series, allows decomposing a single data set into a series of images called ICA components with associated characteristic time-courses, without the need to select a specific temporal profile or constrain the anatomical space of interest. While some of the ICA components describe artifacts and other noise sources in the data, other components represent neurologically meaningful spatio-temporal patterns, which may reflect stimulus-related activations in task-based fMRI or intrinsic functional architecture in task-free fMRI. Particularly, with or without a task, ICA is able to describe the BOLD signal temporal correlations within and between functionally connected brain regions [17]. However, unlike seed-based functional connectivity analysis approaches, the spatial distribution of ICA components does not rely on targeting one or another brain region for the analysis. Moreover, in RS-fMRI, several ICA components correspond to highly reproducible RSNs [3], [5], suggesting how ICA can be used for both characterizing and mapping RSN functional connectivity in a region-independent fashion, and producing separate spatially distributed (voxel-level) representations of distinct mental processes, such as attention and perception, in a task-independent fashion.

Exhaustion is a common psycho-physiological state after prolonged mental performance. It reflects an individual's need for substantial recovery that arises after sustained expenditure of mental effort to meet task demands [18]. Exhaustion is also associated with an increased feeling of fatigue as well as an increase in perceived mental load under unchanged task conditions [19]. Earlier studies have demonstrated that control of attention, monitoring of one's actions and response planning in complex tasks are impaired in relation to the buildup of mental fatigue [20], [21]. These impairments have two potential consequences: either a decrease of performance or an increase of mental effort expenditure to maintain the same level of performance [22]. Moreover, a recent ICA-based cross-sectional study, has reported a higher RS-fMRI activity in all major RSNs, in students scanned at the end of a long period of stress (caused by the preparation for a selection exam), compared to control students scanned during normal academic activities [23], highlighting a possible long-term impact of stress exposure on the resting brain.

The main goal of the present work was to establish and characterize a possible connection between mental exhaustion and the baseline mental activity, as represented by the unconstrained spatial distribution of the ICA-derived RSNs, in an acute phase. To this end, we use RS-fMRI and an ICA-based methodology to investigate the effect of sustained performance over the course of several hours on the RSNs of healthy participants.

## Methods

### 2.1. Ethics statement

The institutional review board for human subject research at the Maastricht University approved the study and all subjects gave written informed consent before the start of the experiment.

### 2.2 Overview of the study methodology

Our RS-fMRI design incorporated both a free day and a working day for all participants to control for circadian effects [24]. In both days RS-fMRI scans were acquired at the beginning and the end of the day at the same daytimes (+/− 30 minutes), corresponding for the working day to a period before the beginning and after the ending of a number of exhausting activities. To maximize the likelihood of inducing exhaustion in all subjects, we used completely unfamiliar tasks, as these normally demand the highest regulative efforts [25]. Specifically, we chose to confront the subjects for the first time with a flight simulator, which is expected to stimulate many concurrent and complex brain activities in a real world scenario.

ICA decompositions were performed on all RS-fMRI scans. The ICA maps from reference scans (free day or beginning of the day) were hierarchically “clustered” within subjects (across days) and between subjects, yielding RSN templates, which were unbiased with respect to the factor of interest. Corresponding RSN components were then evaluated in a second-level analysis to detect any enhancement or suppression effects induced by the sustained mental performance.

In addition to RS-fMRI, we also collected behavioral data at all time points to assess the degree of self-reported exhaustion and verify the possible consequences of exhaustion on cognitive performance and mental effort during a standard N-back task.

### 2.3. Participants

11 healthy participants (mean age 22.7 years, 4 males) were recruited from the Maastricht University student community. In order to minimize potential artifacts in the lower regions of the frontal cortex, we excluded candidates with orthodontic retainers. Screened participants were invited for the testing session.

### 2.4. Tasks and Experimental Procedure

Participants arrived at the lab at the facilities of the Maastricht Brain Imaging Center around 09:30 h, (+− 1 h). Previously, they were instructed to get their normal amount of sleep, and not to exceed moderate caffeine levels in the morning (maximum of two cups of coffee for habitual users not less than one hour pre-experiment). The degree of exhaustion was rated on two 0–150 Visual Analogue Scales (VAS). On one VAS participants were asked to indicate their degree of tiredness, on the other their degree of being rested. Scores of the latter scale were reversed and a mean score of the two scales was calculated.

The participants were then placed in an MRI scanner and the resting state functional and anatomical image data were recorded (see below for MRI parameters). Afterwards, participants performed a version of the N-back task [26]. The task consisted of 15 blocks of 20 N-back trials each, accounting for duration of one minute per block. Participants had to memorize letters appearing on a screen and indicate through a button press response if those letters were identical to the letter one, two, or three trials back, depending on the condition of that block. Each of the three n-back conditions was presented five times, in a quasi-randomized order. Performance was measured as the number of correct button presses within the 2000 ms response window. Participants received feedback after each individual trial in order to be able to continuously adjust their effort expenditure during a block. After each one-minute block, participants rated their subjective expenditure of mental effort on the Rating Scale Mental Effort (RSME) [19]. The task and the RSME were programmed in E-Prime (Psychology Software Tools, Inc., US). They were presented using E-Studio on a Windows XP PC connected to an MRI compatible projector/mirror system. Task and rating input was collected via an MRI compatible optical 2-button joystick (Current Designs Inc., Philadelphia, USA). All subjects had MRI experience and trained the N-back task prior to the experiment, minimizing possible effects of novelty of the environment or learning effects for the task.

After this session, participants either underwent a 4 h practice session in the university's helicopter cockpit mock-up or spent the same amount of time with self-chosen, low-effort activities. The practice session was organized in such a way to induce the exhaustion level of a demanding workday.

The helicopter practice session consisted of a short theoretical instruction on helicopter take-off procedures and a practical part of trying to perform a takeoff procedure according to the presented guidelines. After around 2 h, the session was interrupted to measure the second resting state fMRI scan and a second self-evaluation of exhaustion using VAS.

**Figure 1:**
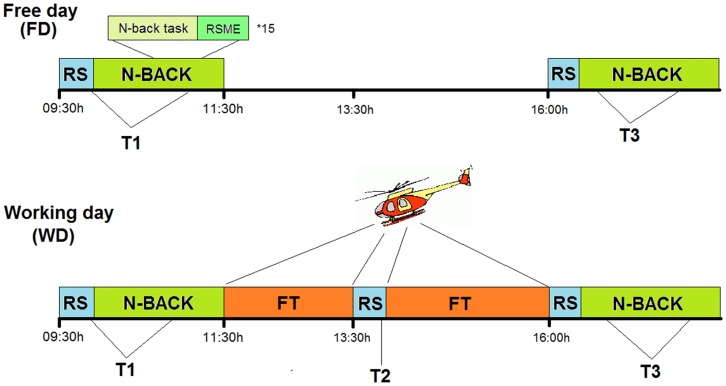
The research design consisted of two days. On the free day, participants were in the lab only in the morning and in the afternoon. In between, they performed self-chosen, low-effort activities. On the working day, morning and afternoon schedules were kept identical, however, an additional resting state measurement was included in the noon. Time scale is not proportional see *methods* for timing information. RS  =  resting state; FT  =  flight task.

After 2 more helicopter practice hours, participants returned to the MRI lab at around 16:00 h. After again indicating their level of exhaustion on the two VAS scales, they were placed back in the scanner. We recorded resting state data with the same parameters and instructions as in the previous sessions. Afterwards, participants performed the same N-back paradigm as in the morning session. All subjects underwent both the free day treatment and the work day treatment in quasi-randomized order. In summary, each subject had five RS-fMRI scans, which for the sake of brevity has been labeled according to the day, free day (FD) or working day (WD) and to the time point (T1, T2 and T3). A graphical description of the study protocol is shown in figure 1.

### 2.5. Behavioral data analysis

Analysis of the behavioral data was conducted in SPSS 18 and 21. Behavioral scores of the two exhaustion scales were pooled after reversion of the “rested” scale. Exhaustion and RSME ratings were z-standardized per participant by subtracting the participant's respective mean and dividing by the participant's respective standard deviation. For the analysis, we only used the ratings at T1 and T3, as the N-back task was only carried out during these two points per day in order to not unduly interrupt the flight task. One participant reported difficulties with the exhaustion scale and therefore data from this participant were excluded from all analyses. For the analysis of the exhaustion scores, we performed a univariate analysis of variance (ANOVA) with time-of-day (morning: T1 versus evening: T3) and the day activity (work day: WD vs free day: FD) as factors. The dependent variable was the normalized exhaustion score. As the results showed a significant interaction of the time of the day and the day activity, we subsequently contrasted the scores per level of daytime and day activity. A Bonferroni adjustment for multiple comparisons was applied to the p-values of the simple main effects.

For the effort data, an initial exploratory general linear model (GLM) of effort scores at T1 using day activities and N-back conditions as factors showed an unexpected effect of day activity at T1. We suspected that this might show learning effects for the N-back tasks. Due to exclusion of 3 participants (see above in this section and section 2.7), the order of the working day and the free day were not balanced, as 6 of the remaining 8 participants started with the working day. We thus included the order of test days (first versus second day) as covariate. The subsequent GLMs that we calculated separately for T1 and T3 thus included day activities and N-back conditions as factors and the order of test days as a covariate.

An influence of our treatment on the error counts made on the N-back task was tested using a GLM per time of day with N-back condition and day activities as factors and order of test days as covariate, assuming a negative binomial distribution [27]. All p-values from multiple comparisons were adjusted using the Bonferroni correction.

### 2.6. MRI and fMRI data acquisition

The participants were placed in a Siemens Allegra 3T head scanner (Siemens AG, Erlangen, Germany). Respiratory and pulse measurement devices in the form of an expandable breath sensor and an infrared finger clip were fitted on the participants.

The resting state data were recorded with the participant's eyes closed and the instruction to stay awake, but to not engage in any specific mental activity for the time of the scan. Whole brain Echo-Planar Imaging (EPI) was performed using the following parameters: Matrix size 64×64; slice thickness 3 mm; Slice order descending and interleaved; no gap; FOV 192×192 mm; TE = 30 ms; TR = 2000 ms, 180 repetitions. Slice orientation was tilted in order to minimize susceptibility artifacts in the orbitofrontal regions [28]. Anatomical imaging was carried out with a standard Alzheimer Disease Neuroimaging Initiative (ADNI) complaint T1 weighted sequence, voxel size1 cubic mm; flip angle  = 9 deg; TR = 2250 ms; TE = 2.6 ms.

### 2.7. MRI and fMRI data preparation

Standard (f)MRI image data preparation, normalization and pre-processing and statistical analysis and visualization were performed with the BrainVoyager QX software (Brain Innovation B.V., The Netherlands) [29]. Functional data preprocessing included the correction for slice scan timing acquisition, the 3D rigid body motion correction and the application of a temporal high-pass filter with cut-off set to 2 cycles per time-course. One participant showed excessive (>4 mm) movement in at least one of the scanning runs. Additionally, data from one run of another participant were lost due to a file transfer error. Data from both of these participants were excluded from all analyses. Structural and functional data of the remaining 8 subjects were co-registered and spatially normalized to the Talairach standard space. In the course of this procedure, the functional images were resampled to an isometric 3 mm grid covering the entire Talairach box.

To account for possible BOLD effects due to cardiac pulsation and respiratory cycle [30] physiological noise correction was performed on each functional scan using the RETROICOR technique [31]. Time-courses for components of heart rate, respiration and respiration volume per time were created from the recorded physiological signals at the fMRI sampling rate using Matlab scripts (The Mathworks, United States) available from the AFNI suite [32] and used, together with the motion estimate time-courses available from the previous 3D rigid body motion correction, as predictors in single-study GLM analysis [33] of each functional scan. Using the residual time-courses from this GLM allowed us to regress out possible signal fluctuations time-locked with the phase of cardiac and respiratory cycles and residual movement-related signal fluctuations.

### 2.8. Resting-state fMRI data analysis

Single-subject and group-level ICA analyses were performed on the pre-processed functional time series and the estimated independent components using two plug-in extensions of BrainVoyager QX implementing the fastICA algorithm [34] and the self-organizing group-level ICA (sog-ICA) algorithm [35], [36].

For each subject and each scan, 30 independent components were extracted and scaled to spatial z-scores (i.e. the number of standard deviations of their whole-brain spatial distribution). These values express the relative amount a given voxel is modulated by the activation of the component [17] and hence reflect the amplitude of the correlated fluctuations within the corresponding functional connectivity network. The final number of ICA components is a free parameter, which has previously been either empirically determined or estimated [37] as the number of principal components retained in the multivariate data. This number typically lies between 20 and 60 depending on the data. In the present work we used the same rule of thumb of Grecious et al. [38] and chose to keep a number of principal components corresponding to at least one sixth of the number of time points (180/6 = 30) and accounting for more than 99.9% of the total variance.

The group ICA analysis was conducted in two steps.

In the first step, the baseline scans from both days (FD-T1 and WD-T1) were submitted to a hierarchical sog-ICA analysis [38]. Thereby, the most representative RSN maps were selected for the second step. To minimize day- and treatment bias, the baseline scans of the two days were pooled together in the first level of the hierarchical sog-ICA and the scans corresponding to the exhausted states were not included. The final result of the first step consisted of 30 clusters of 8 components (one per subject) and this was the basis of a random-effects analysis, which was conducted as a 1-factor ANOVA with 1 within-subject factor (“cluster membership”) and subjects as random observations. From this ANOVA, we produced 1-sample t-test maps (one for each cluster) and identified at least seven of the most relevant RSN components as reported and illustrated in previous studies (see, e. g., [5]). For each identified RSN, an RSN template mask was obtained by applying a voxel-level threshold of P = 0.05 (corrected for multiple voxel-level comparisons).

In the second step, all RSN template masks were used to select one best-fitting component per subject per RSN in each separate scan [39], [40]. Thus, for each RSN, we obtained five best-fitting RSN components per subject (one component per condition), and then submitted 32 individual components (4 components per condition: FD/WD_T1/T3, 8 subjects) to a random effects 2-factor ANOVA with 2 within subject factors: “day” (F vs W) and “time point (“T1 vs T3”). The components corresponding to the intermediate condition WD_T2 were not used in the 2-way ANOVA, but were considered for display purposes in the subsequent regional analyses.

In order to isolate exhaustion-induced effects, from this 2-way ANOVA, we combined three linear contrasts, respectively accounting for (i) the effect of the treatment across days at T3: [WD-T3>FD-T3], (ii) the effect of treatment in the working day: [WD-T3>WD-T1] and (iii) the effect of treatment across days and time points [WD-T3>FD-T1]. The third contrast was added to exclude any interaction effects implied by the contrast [FD-T3<FD-T1] and not by the contrast [WD-T3>WD-T1]. Combining all these inequalities yields: [3*WD-T3>WD-T1+FD-T1+FD-T3], which is the linear contrast that we have tested to identify the regions with statistically significant effects of “enhancement” or “suppression” of the regional RS-fMRI oscillations. To correct for multiple comparisons, statistically significant regional effects from this contrast were only accepted for compact clusters surviving the joint application of a voxel- and a cluster-level threshold, which were chosen using a non-parametric randomization approach. Namely, an initial uncorrected threshold was applied (p = 0.01) to all voxels and, then, a minimum cluster size was calculated that protected against false positive clusters at 5% after 500 Monte Carlo simulations [29], [41]. To further correct for the number of studied RSNs, a cluster-corrected level of 5/N% was considered, N being the number of RSNs selected in the first step of the analysis (and therefore the number of actual 2-ANOVA contrasts).

For regions of interest determined in the above analysis, individual ICA z-scores were extracted for the scans WD-T2 and WD-T3, averaged over all voxels and used for a linear correlation analysis with the degrees of exhaustion, as expressed by the mean VAS score. To compute a pooled statistical significance of these correlations, while correcting for the implicit study effect, we entered these regional ICA scores into a 2-way analysis of covariance (ANCOVA) with one categorical factor (scan), one continuous factor (VAS score) and a scan-by-exhaustion interaction term. Because these analyses were only exploratory, we report their statistical significance level without correction for multiple comparisons.

## Results

### 3.1. Behavioral results

**Figure 2:**
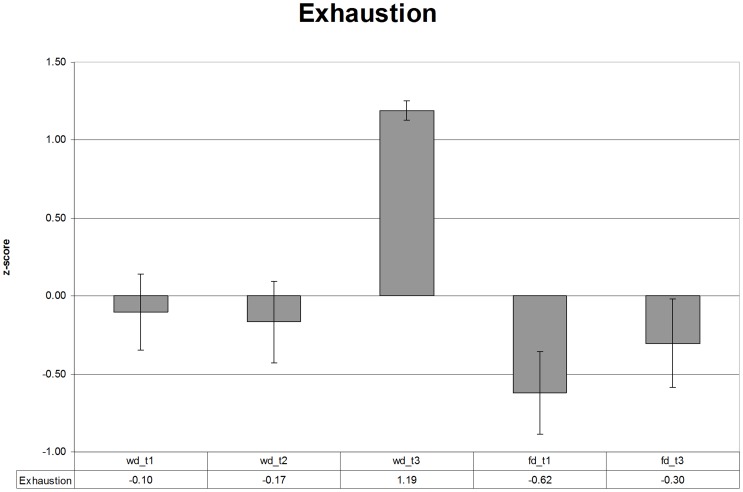
Exhaustion levels over the course of the work (wd_t1/t3) and the free day (fd_t1/t3) with standard error bars.

In the analysis of the exhaustion scores, the results of the ANOVA showed that both treatment (*F* (1,28) = 21.66, *p*<0.001) and time-of-day (*F* (1,28) = 13.85, *p*<0.001) had an influence on the level of exhaustion. There was a significant interaction effect of time-of-day and the day activity (*F* (1, 28) = 5.6 *p* = 0.032). Analysis per level of time-of-day showed that exhaustion was rated higher (*F* (1, 28) = 23.84, *p*<0.001) in the afternoon of the work day (WD-T3) than on the afternoon of the free day (FD-T3). There was no significant difference at the T1 measurement points (*F* (1,28) = 2.89, *p* = 0.1) (see Figure 2).

**Figure 3:**
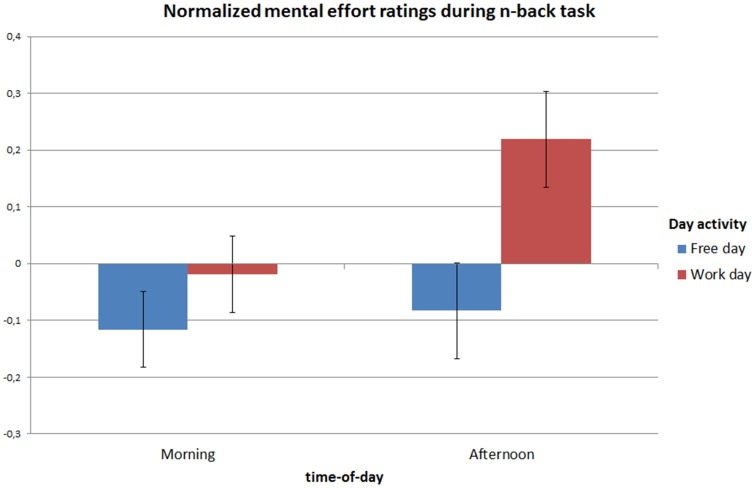
Effort ratings for all N-back conditions as an effect of treatment (work vs free days): Estimated marginal means per time-of-day and day activity (value of the centered covariate day order  = 0). Error bars represent standard errors.

When testing for differences in RSME ratings at T1, we initially found an unexpected effect of day activity (*F* (1,236) = 18.98, *p*<0.001). As we suspected a possible learning effect, we repeated the analysis with the order of test days as a covariate. The covariate test day indeed had a significant influence on the RSME scores (*F* (1,235.) = 38.46, *p*<0.001). The factorN-back condition was still significant (*F* (2,235.) = 107.91, *p*<0.001), while day activity was no longer a significant factor (*F* (1,235.) = 0.92, *p* = 0.338). At T3, still both the covariate order of test days (*F* (1,235.) = 10.89, *p*<0.001) and the factor N-back condition (*F* (2,235.) = 26.53, *p*<0.001) were both significant. At this point, however, also day activity was significant (*F* (1,235.) = 5.60, *p* = 0.0192) (see Figure 3).

All participants were able to perform the N-back task without problems, as the average error rates per condition suggest (1-back: mean  = .81, SD  = .95; 2-back: mean  = 1.22, SD  = 1.39 and 3-back: mean  = 2.49, SD = 2.02). The performance of the participants, measured in number of errors, was shown to be influenced at T3 by both the order of test days (Wald *χ^2^* (1, *N* = 240) = 7.89, *p*<0.005) and the N-back condition (Wald *χ^2^* (2, *N* = 240) = 14.87, *p*<0.001), but not the day activity (Wald *χ^2^* (1, *N* = 240)  = .96, *p* = 0.326).

### 3.2. Imaging results

**Figure 4:**
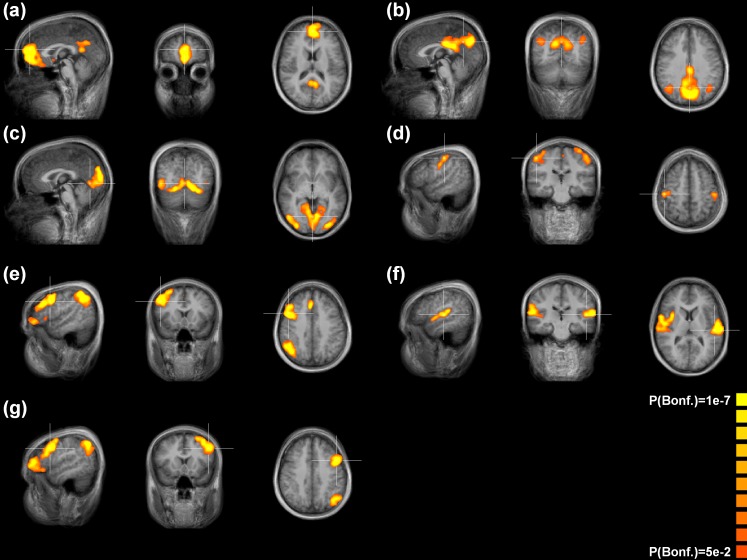
Group ICA results for the analysis of the pooled baseline resting state scans (WD-T1, FD-T1): (a) anterior default mode network; (b) posterior default mode network; (c) visual network; (d) sensory-motor network; (e) right fronto-parietal network; (f) auditory network; (g) left fronto-parietal network. Statistical thresholds were corrected for multiple comparisons.

In the first step of the analysis including only the morning sessions, at least seven RSN components were identified that were highly similar to those reported in previous ICA-based RS-fMRI studies. These RSN components could be functionally categorized by the Talairach coordinates of the most active sub-regions (for reference, we used, e. g., the table presented by Allen et al. [42]) and were accordingly labeled as (two) default-mode networks (an anterior and a posterior default-mode network), a visual network, an auditory network, a sensori-motor network, and two (lateralized) dorso-lateral fronto-parietal networks. These components were selected as “baseline” RSN components for the second step of the analysis (fig. 4).

In the second step of the analysis, that included both free and work sessions, clusters of voxels displaying statistically significant differences in the comparison between the most exhausted condition and the mean of all other conditions were detected within four RSNs. These are reported in table 1 and will be illustrated and discussed below.

**Table 1:** Regional effects of exhaustion in resting-state networks (individual clusters).

Resting-state Network	Anatomical Region	Center of Mass x,y,z (Talairach)	T-stat [Avg, Max]	Extension [mm^3^]	Correlation with Exhaustion [F-stat, p-value]
Early Visual	Left Lingual Gyrus	−16, −62, 0	+4.16, +6.77	1796 (**)	6.7, 0.0237 (***)
Anterior Default-mode	Medial Frontal Gyrus	−1, +46, +33	+4.06, +6.24	380 (*)	6.04, 0.03 (***)
Left Fronto-Parietal	Left Middle Frontal Gyrus	−34, +7, +48	−4.59, −9.82	2590 (**)	0.01, 0.93
Right Fronto-Parietal	Right Angular Gyrus	+46, −61, +36	−3.50, −7.369	887 (**)	0.57, 0.46
Right Fronto-Parietal	Right Middle Frontal Gyrus	+33, +7, +35	−4.24, −6.568	911 (**)	2.52, 0.13

(*) P<0.05 cluster-level corrected.

(**) P<0.05 cluster- and network-level corrected.

(***) Uncorrected p-value.

**Figure 5:**
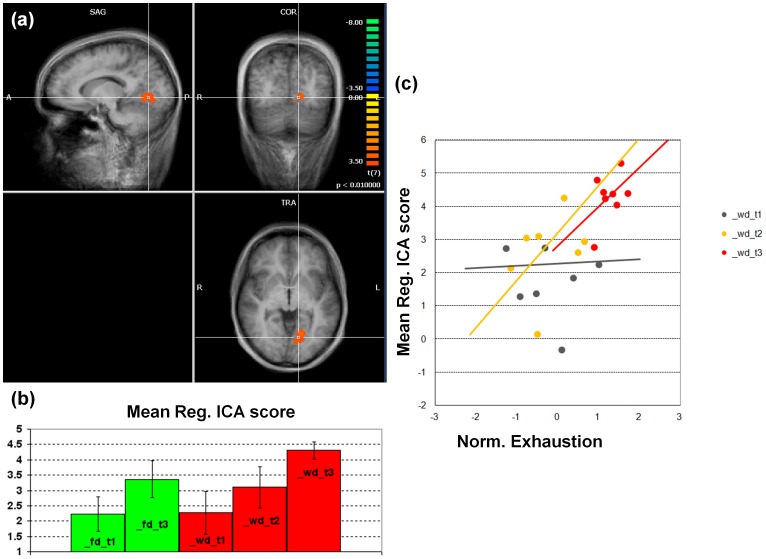
Early visual processing network. (a) Statistical map of the differential effects (WD_T3>WD_T1+FD_T1+FD_T3). Statistical thresholds were corrected for multiple comparisons. (b) Bar graph of the regional ICA scores (with standard error bars) in all conditions. (c) Correlation graph of the regional ICA scores against the normalized degree of exhaustion for the separate working day sessions (with fit lines indicating the directions of the correlations).

**Figure 6:**
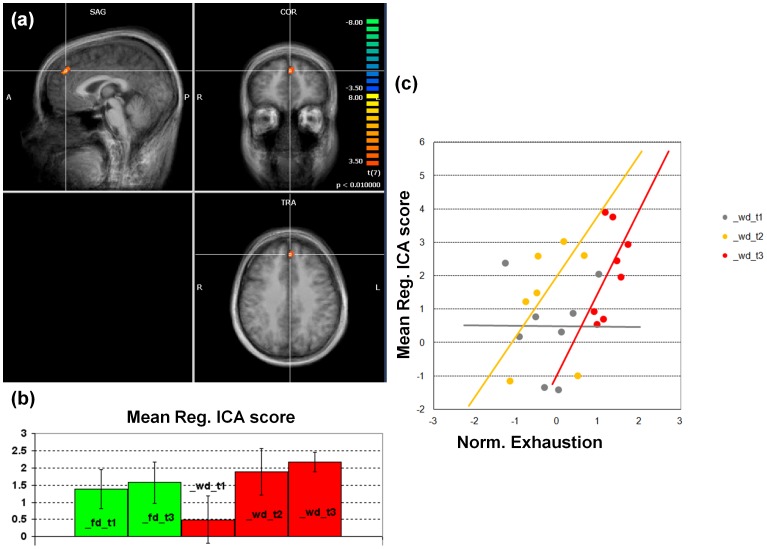
Anterior default mode network. (a) Statistical map of the differential effects (WD_T3>WD_T1+FD_T1+FD_T3). Statistical thresholds were corrected for multiple comparisons. (b) Bar graph of the regional ICA scores (with standard error bars) in all conditions. (c) Correlation graph of the regional ICA scores against the normalized degree of exhaustion for the separate working day sessions (with fit lines indicating the directions of the correlations).

**Figure 7:**
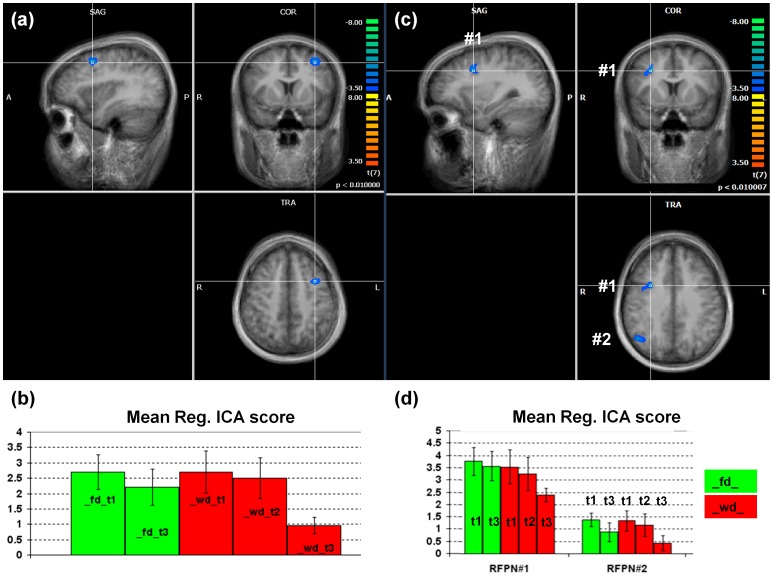
Left (a,b) and right (c,d) fronto-parietal networks. (a,c) Statistical maps of the differential effects (WD_T3>WD_T1+FD_T1+FD_T3). Statistical thresholds were corrected for multiple comparisons. (b,d) Bar graphs of the regional ICA scores (with standard error bars) in all conditions.

We found statistically significant regional effects of exhaustion in the early visual processing network (fig. 3), in the anterior default mode network (fig. 4) and in both the right (fig. 5) and the left (fig. 6) dorso-lateral fronto-parietal networks(fig. 7), corresponding to the two executive attention networks.

In the visual processing network (fig. 5), a compact cluster located in left lingual gyrus showed a statistically significant regional effect, with rs-fMRI signals enhanced in the most exhausted condition (p<0.01, clus. size >702 mm^3^, cluster- and network-level corrected). Extracting the average ICA scores in this region from each individual revealed a statistically significant positive correlation with the self-reported exhaustion scores (p = 0.0237) but no significant scan-by-exhaustion interaction (p>0.05).

We found no significant effects in the anterior and posterior default mode networks. At an uncorrected level of significance, in the anterior default mode network, a compact cluster located in medial frontal gyrus showed an effect similar to that observed in the early visual processing network (p<0.01, clus. size >378 mm^3^, cluster-level corrected) with a positive correlation between the regional ICA scores and the exhaustion scores (p = 0.03) (fig. 6) and no significant scan-by-exhaustion interaction (p>0.05).

Finally, both left and right fronto-parietal executive attentional networks exhibited reduced functional connectivity in the most exhausted state compared to all other states (fig. 7). More specifically, in the left fronto-parietal executive attention network, a compact cluster located in left middle frontal gyrus showed an effect opposite to that observed in the anterior default mode and the visual processing networks (p<0.01, clus. size >675 mm^3^, cluster- and network-level corrected). In the right fronto-parietal network, the suppression of RS-fMRI signals was distributed across the anterior and posterior nodes. In fact, two compact clusters (p<0.01, clus. size >540 mm^3^, cluster- and network-level corrected) located anteriorly in right middle frontal gyrus and posteriorly in the right angular gyrus exhibited a similar significant suppression. However, for none of the regions, the correlation with behavioral scores or the scan-by-exhaustion interaction reached statistical significance (p>0.05).

## Discussion

In this study, we intended to explore the distributed topological changes of the main RSNs in healthy volunteers who spent an entire full day in completing challenging and demanding tasks. By using a multi-level ICA-based approach, we demonstrated domain-specific spatially selective effects, which were in some cases correlated with the increased levels of mental exhaustion reported by the studied subjects.

### 4.1 Behavioral effects of mental exhaustion

The behavioral results were in line with our hypotheses. Namely, the sustained performance in the working day induced an increased self-reported level of exhaustion compared to the free day.

In the working day, there was no significant difference between exhaustion levels at T1 and T2. We speculate that the short training periods were not enough to actually exhaust the participants strongly already at T2. This reflects our rationale to design a paradigm featuring longer task duration. In fact, a short period of activity in a stimulating environment such as a flight simulator can be assumed to actually increase arousal and thereby decrease experienced exhaustion. Also, it is possible that at T1, participants still experienced effects of sleep inertia and low body temperature, as their bodies just started to wake up (see, e. g., [43]). Nonetheless, when correlating brain and behavior, we pooled data from both T2 and T3 as we did not want to exclude the possibility that functional brain networks could predict potential sub-threshold exhaustion effects already at T2.

Next to an increase in exhaustion, we also found a significant increase of mental effort expenditure to maintain the same performances (i. e. same error rates) in the N-back task at the end of the working day compared to the free day at identical day times. This means that participants were able to sustain their performance level, yet they had to invest a significantly higher amount of mental effort. This finding is consistent with the framework of Hockey [22] and similar findings have been demonstrated earlier (see, e. g., [21]).

### 4.2 Distributed effects of mental exhaustion on the early visual processing network

A remarkable effect of the work treatment was detected in the early visual processing RSN, where the RS-fMRI fluctuations were significantly higher in the exhausted state and the regional scores were positively correlated with the degree of exhaustion. This RSN has been previously found to be principally associated with the electro-encephalographic (EEG) “alpha” rhythm [5], which is typically more prominent in exhausted states, such as after sustained visual attention [20] or driving tasks [44], [45], but also proportionally more attenuated in relaxed states, such as after a daytime nap [46]. Most relevant to our findings, a recent study by Lim et al. [47] has demonstrated how EEG alpha activity can even predict successful recovery from a sustained mental task if measured during a short break: Namely, participants showing a lower level of EEG alpha activity in a resting period between two blocks of task performance also showed a higher increase in performance after the break. Taking these evidences together, and considering that it is generally believed that the presence of alpha oscillations signifies “idling” or inhibition of cortical processing [48], [49], the observed regional increase of the RS-fMRI signal fluctuations in the visual network induced by exhaustion clearly points to increased inhibition of the neural activity in the early visual system implied by exhaustion.

### 4.3 Distributed effects of mental exhaustion on the default-mode networks

Albeit only descriptively, we also report a weak positive effect of exhaustion in the anterior DMN, characterized by the regional increase of RS-fMRI signal fluctuations in the medial frontal gyrus. In this region, the correlation between the mean regional ICA scores and the self-rated degree of exhaustion was also positive and statistically significant in the exhausted states. A similar effect of increased resting state frontal connectivity in the anterior DMN has been recently observed in patients with mild traumatic brain injury [50]. In contrast to this study, however, no significant effects were found in the posterior DMN.

### 4.4 Distributed effects of mental exhaustion on the fronto-parietal networks

Different from what has been observed in the early visual processing and the anterior default mode networks, both the right and the left dorso-lateral fronto-parietal networks were found to be suppressed in the exhausted, compared to the relaxed states, with clusters of significantly negative differences in frontal and parietal lateralized regions.

The suppression of the fronto-parietal networks could be a sign that brain fatigue is deteriorating the subjects' executive abilities. In fact, the suppressed areas in these networks have long been established as crucial in the effortful maintenance of sustained attention [51], [52], [53], [54], [55], [56], therefore the functional connectivity of these networks could be impaired after long periods of sustained activation with associated heavy mental workload. In our experimental and analytical framework, this possible RSN impairment was detected before performance degradation in the subsequent N-back tasks, as demonstrated by the same error rates at the end of the working and free days.

The suppression of fronto-parietal networks observed with RS-fMRI in a significantly exhausted state could be linked to recent arterial spin labeling (ASL) perfusion based fMRI (P-fMRI) results [53], [56].

Lim et al. [56] have showed that sustained periods of taxing cognitive workload represented by 20-min executions of a psychomotor vigilance test (PVT), besides measurable performance declines, also cause significant deactivations (as reflected by regional cerebral flow (rCBF) reductions) in a right fronto-parietal network during a post-task resting interval, compared to a pre-task resting baseline. Remarkably, it is this rCBF decrease between post- and pre-task baselines, and not the rCBF increase during the task, that turns out to predict the PVT performance decline, suggesting the presence of persistent effects of cognitive fatigue in the right fronto-parietal network after a period of heavy mental work, as well as the critical role of this executive attention network in mediating the (temporary) impairment of cognitive abilities.

Even if RS-fMRI suppression occurred in both dorsal fronto-parietal networks, this effect appeared more widespread and distributed over distant regions in the right lateralized network, where both frontal and parietal nodes were suppressed, than in the left dorsal fronto-parietal network, where only one anterior region in the left middle frontal gyrus was suppressed. In other words, the suppression appears to be a more generalized phenomenon in the right fronto-parietal network, even if the correlation with the self-rated degree of exhaustion does not reach statistical significance in any of the single regions with detected differences. Contrariwise, the suppression is stronger in the left fronto-parietal network, suggesting a potentially worse damage in the anterior portion of this network.

In line with the idea that more sustained activity during the tasks causes more deterioration to the executive attention network after the tasks, the more widespread involvement of the right fronto-parietal network could be linked to the right lateralization reported (in both human and monkey studies) when these areas are engaged in tasks requiring attention, vigilance and continuous performance [51], [57], [58], [59], [60], [61], [62]. In the working day, i.e. during intensive helicopter flying practice in the simulator, subjects were confronted with a task that required continuous attention and careful control of the cockpit interfaces to successfully operate the aircraft. Hence, the task requirements in our study can be assumed to strongly engage the right fronto-parietal network, even if we cannot be as specific as to predict which aspect of the task in particular is responsible for the reported increase of exhaustion and the suppression of the right fronto-parietal network. Contrariwise, the higher deterioration observed in the left middle frontal gyrus (compared to its contra-lateral counterpart) could be attributed to the fact that in healthy (non-depressed) subjects cognitive performance inducing stress, causes abnormally higher activations in left compared to the right middle frontal gyrus during the same working memory task [63]. Thereby, it is plausible that this kind of stress could have contributed to the more regionally specific deterioration of the left fronto-parietal network.

Taken together, our results demonstrate mental exhaustion affects RS-fMRI activity in different directions depending on the RSN. Namely, visual and default-mode networks exhibit a local up-regulation, and fronto-parietal networks exhibit a local down-regulation, of network-specific RS-fMRI activity. These findings suggest that mental exhaustion affects RSNs not depending on the character of being a task-positive or a task-negative network (which would, e.g., imply an opposite regulation of visual and default-mode networks) but, more likely, on the character of being associated with sensory inhibition or excitation (as is the case for the visual network) and on the character of being associated with intrinsic or extrinsic neural processes (as is the case for default-mode and the fronto-parietal networks).

Moreover, when correlating regional ICA scores with exhaustion levels, significant coupling were found in the upregulated, but not in the downregulated, RSNs. A likely explanation for this finding is that RSN upregulation implies higher signals, and, therefore, a higher signal-to-noise ratio, in the “exhausted” states, and this produces less noisy observations in the post-hoc correlations. Contrariwise, RSN downregulation causes relatively more noisy observations in the exhausted states. Thus, we could expect more statistical power in correlating the upregulations than the downregulations.

### 4.5 Relation to previous works

Other groups have previously addressed possible alterations in RSN functional connectivity and their endogenous dynamic features that can be related to cognitively effortful tasks. For instance, using a similar “rest-task-rest” design, Barnes et al. [64] found that brain endogenous dynamics tend to recover some of their pre-task dynamic features relatively slowly, suggesting that large-scale neurocognitive systems can take a considerable period of time to return to a stable baseline state after demanding task. In line with this theory, Gordon et al. [65] demonstrated that after a working memory task, the immediately following resting state functional connectivity remained persistently altered compared to the baseline resting state, in a fashion similar to how this was altered during the task performance. Particularly, when examining the task positive network (TPN) and the DMN, a suppression of the within-network functional connectivity in the TPN (as well as a modest non-significant enhancement of the DMN) was observed in this study, which seems highly relevant to the interpretation of the present findings. In fact, following Gordon et al. [65], the observed reduction of the functional connectivity in the fronto-parietal networks may well reflect a cognitive after-effect, such that the brain continues to ruminate with the flight simulation after the task is ceased, or, more likely, the persistence of subjective aspects from the recent effortful cognitive experience. This is possible because fronto-parietal activity is associated with diverse cognitive tasks and demands that are central to intelligent thoughts, actions and behaviors, including the creation of structured mental programs, the control and (rapid) reorganization of the mental focus and the separation of successive task steps [66], all problem-solving abilities stimulated by the flight simulator.

Some studies examining pre vs post task resting state functional connectivity have also suggested or demonstrated the existence of learning effects in multiple domains, albeit not all in the same RSNs and regions, and not all in the same direction (i. e. manifesting as positive or negative changes). Duff et al. [67] have shown negative changes in the sensorimotor network following a simple finger tapping exercise. Using motor learning paradigms, Albert et al. [11] have reported an increased functional connectivity in the left fronto-temporal network, whereas Vahdat et al. [68] found that the only regions showing functional connectivity changes purely associated with motor learning were located in the cerebellar cortex and in the superior parietal lobule and all exhibited negative correlations with learning. Using a visual learning paradigm, Lewis et al. [69] were in line with Albert et al. [11] in that within-network learning effects would mainly consist of increased resting state functional connectivity that were related to highly specific synaptic mechanisms stimulated by the learning task. Finally, both Waites et al. [70] and Grigg and Grady [71] used different language tasks to show a positive after-effect of the cognitive activity in which positive changes in resting connectivity were prevalent in high order areas. The common denominator of all these evidences is that learning effects generally cause positive changes in the resting state functional connectivity of high order brain regions and networks, whereas low order domains, such as the sensorimotor or visual domains, might require highly specific learning tasks to show similar learning-related effects prevailing over perceptual or fatigue-related effects. Along this line, considering that our stimulation was not specific to any of the studied low order systems (but rather engaged all brain systems in highly and purposely effortful performances), and that we observed a reduction of the resting state functional connectivity in both the left and the right FPN, we suggest that the learning factor was not as prominent in our experimental design as the exhaustion factor.

### 4.6 Limitations

This study has two important limitations. First, given the low number of subjects, the reported analyses are to be considered only exploratory and, therefore, all results are at present useful only for elaborating initial working hypotheses in the design of future studies. Second, it should be considered that the N-back task, while very common and popular in cognitive experiments, could be not appropriate in relation to possible learning effects induced by the practice with the flight simulator. In our experiments, it was not possible (due to technical problems) to record objective indicators regarding the performance at the simulation and therefore the possibility that the extensive practice with the simulator might also have caused that subjects gain knowledge of new skills, cannot be excluded. On the other hand, several studies have shown that apparently less efficient mental processes (resulting, e. g., in increased reaction time on task) can actually correspond to improved performances due to practice (see, e. g., [72]).

## Conclusions

In conclusion, this study demonstrates the potential utility of RS-fMRI, in combination with ICA-based distributed modeling of the spontaneous BOLD activity synchronization, for revealing and characterizing the neural networks that present some immediately visible effects of mental exhaustion after sustained and highly demanding training and practice. In fact, our results demonstrate that some of the ICA components corresponding to the most important and reported RSNs exhibit regional changes in their spatial distribution which can be associated with persistent effects of exhaustion, as measurable in the immediate aftermath of a period of heavy cognitive workload. Therefore, characterizing the resting state functional connectivity of these RSNs with ICA may provide novel markers of cognitive fatigue and mental exhaustion useful for identifying neural “risk factors” for accidents and errors due to prolonged task performance. For instance, considering the specific paradigm used here, this framework could be potentially useful to identify risk factors in pilots in relation to, e. g., the maximum number of flight hours allowed. Moreover, a possible clinical implementation of this paradigm could help neurologists to address the effects of cognitive fatigue in neurological diseases [73].
